# A small molecule inhibitor of dengue virus type 2 protease inhibits the replication of all four dengue virus serotypes in cell culture

**DOI:** 10.1186/s12985-015-0248-x

**Published:** 2015-02-08

**Authors:** Rajendra Raut, Hemalatha Beesetti, Poornima Tyagi, Ira Khanna, Swatantra K Jain, Variam U Jeankumar, Perumal Yogeeswari, Dharmarajan Sriram, Sathyamangalam Swaminathan

**Affiliations:** Recombinant Gene Products Group, International Centre for Genetic Engineering and Biotechnology, New Delhi, 110067 India; Department of Biotechnology, Jamia Hamdard, Hamdard Nagar, New Delhi, 110062 India; Department of Biological Sciences, Birla Institute of Technology and Science Pilani, Hyderabad Campus, Hyderabad, 500078 India; Department of General Medicine, Lady Hardinge Medical College, Shaheed Bhagat Singh Marg, New Delhi, 110001 India; Department of Pharmacy, Birla Institute of Technology and Science Pilani, Hyderabad Campus, Hyderabad, 500078 India; Centre for Infectious Disease Research, Birla Institute of Technology and Science Pilani, Hyderabad Campus, Hyderabad, 500078 India

**Keywords:** Dengue fever, Dengue virus, NS2b-NS3 protease, Dengue protease inhibitor, Antiviral therapy

## Abstract

**Background:**

Dengue has emerged as the most significant of arboviral diseases in the 21st century. It is endemic to >100 tropical and sub-tropical countries around the world placing an estimated 3.6 billion people at risk. It is caused by four genetically similar but antigenically distinct, serotypes of dengue viruses. There is neither a vaccine to prevent nor a drug to treat dengue infections, at the present time. The major objective of this work was to explore the possibility of identifying a small molecule inhibitor of the dengue virus protease and assessing its ability to suppress viral replication in cultured cells.

**Methods:**

We cloned, expressed and purified recombinant dengue virus type 2 protease. Using an optimized and validated fluorogenic peptide substrate cleavage assay to monitor the activity of this cloned dengue protease we randomly screened ~1000 small molecules from an ‘in-house’ library to identify potential dengue protease inhibitors.

**Results:**

A benzimidazole derivative, named MB21, was found to be the most potent in inhibiting the cloned protease (IC_50_ = 5.95 μM). *In silico* docking analysis indicated that MB21 binds to the protease in the vicinity of the active site. Analysis of kinetic parameters of the enzyme reaction suggested that MB21 presumably functions as a mixed type inhibitor. Significantly, this molecule identified as an inhibitor of dengue type 2 protease was also effective in inhibiting each one of the four serotypes of dengue viruses in infected cells in culture, based on analysis of viral antigen synthesis and infectious virus production. Interestingly, MB21 did not manifest any discernible cytotoxicity.

**Conclusions:**

This work strengthens the notion that a single drug molecule can be effective against all four dengue virus serotypes. The molecule MB21 could be a potential candidate for ‘hit-to-lead’ optimization, and may pave the way towards developing a pan-dengue virus antiviral drug.

**Electronic supplementary material:**

The online version of this article (doi:10.1186/s12985-015-0248-x) contains supplementary material, which is available to authorized users.

## Background

Dengue is an arboviral disease which is currently a very significant global public health concern [1-3]. The disease is endemic to >100 tropical and sub-tropical countries. Of the ~3.6 billion people estimated to be at risk of dengue, ~400 million people experience dengue infections annually [4]. Four antigenically distinct serotypes of dengue viruses (DENV-1, −2, −3 and −4) of the genus *Flavivirus*, family *Flaviviridae*, cause this disease [5]. Clinically, the disease has been distinguished as either mild dengue fever (DF) or potentially fatal dengue hemorrhagic fever (DHF) and dengue shock syndrome (DSS) [6]. Despite decades of efforts, a preventive dengue vaccine is not available [7,8]. Ongoing efforts have revealed the existence of challenging hurdles in dengue vaccine development [9-11]. This has spurred attention towards exploring the feasibility of developing therapeutic drugs [12,13]. Observations that the virus titers in DHF/DSS patients are an order of magnitude higher in comparison to DF patients [14,15], suggest that a drug which can bring about ~1 log reduction in virus replication may be able to prevent the progression of DF to DHF/DSS.

The DENV genome is a single-stranded positive sense, ~11 kilobases (Kb) long RNA molecule [5]. It carries a 5’ cap, but no poly A tail and contains a single large open reading frame (ORF) sandwiched between two non-translated regions located at either end. The ORF is translated in the infected host cell cytoplasm into >3000 amino acid (aa) residue long polyprotein precursor. Co- and post-translational processing of this precursor by host and viral proteases generates ten viral proteins, of which three are structural and the rest, non-structural (NS) proteins [5]. One of these latter proteins, NS3, by virtue of its function as the viral protease is crucial in the polyprotein maturation process [16]. The protease activity of NS3, located in the N-terminal one-third of the full-length molecule, contains the classic catalytic triad seen in serine proteases and relies on cofactor function, mapping to a hydrophilic 40 aa residue domain of another viral protein, NS2b [17]. Mutations in either component of the flaviviral protease that compromise its function lead to abrogation of replication [18,19]. This two component protease, NS2b-NS3Pro, has emerged as a potential antiviral drug target in recent years [16].

Ideally, a DENV inhibitor must be effective against all four DENV serotypes. This is because, each of the four DENVs can cause the full spectrum of dengue disease, and all four DENV serotypes tend to co-circulate in hyperendemic regions [2,3]. Functional profiling studies indicate that the NS2b-NS3Pro of the four DENV serotypes share very similar peptide substrate structure activity relationships [20]. Based on this notion, we have explored the feasibility of identifying an inhibitory molecule with pan-DENV-specificity by empirical screening of an ‘in-house’ library of ~1000 small molecular weight compounds. This paper presents the identification of a DENV-2 NS2b-NS3 protease (NS2b-NS3Pro)-inhibitory molecule which could inhibit the replication of all four DENV serotypes in infected cells in culture.

## Results and discussion

### Recombinant DENV-2 NS2b-NS3Pro

**Figure 1:**
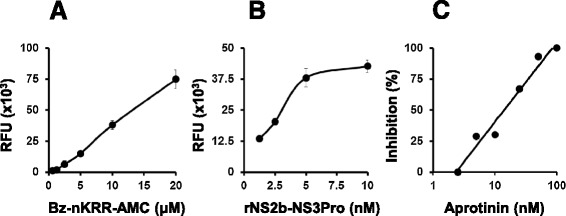
**DENV-2 NS2b-NS3Pro enzyme assay, optimization and validation. (A)** Kinetics of NS2b-NS3Pro action as a function of substrate concentration (at 5nM enzyme). **(B)** Rate of enzyme catalysis as a function of enzyme concentration (at 10 μM substrate). **(C)** Activity of the cloned NS2b-NS3Pro as function of aprotinin concentration (5nM enzyme, 10 μM substrate, 20 min incubation). Activity in the absence of aprotinin was taken as 100% (RFU = relative fluorescence units).

We cloned and expressed DENV-2 protease in *E. coli* and purified it to >90% homogeneity using modifications of previously reported methods [20-22]. The design of a synthetic *NS2b-NS3Pro* gene, its expression in *E. coli* and its purification by Ni^2+^-NTA affinity chromatography are summarized in Additional file 1: Figures S1 and S2. Using the synthetic fluorogenic peptide Benzoyl-Nle-Lys-Arg-Arg-4-methylcoumarin-7-amide (Bz-nKRR-AMC), which has been shown to be a better substrate compared to peptides containing endogenous dengue cleavage sites [20], we confirmed that our purified DENV-2 NS2b-NS3Pro is enzymatically active based on the increase in fluorescence that accompanies peptide cleavage (Figure 1). Assay conditions were optimized to identify enzyme and substrate concentration ranges compatible with a linear dose–response (Figures 1A, and B). To validate this assay for inhibitor screening, we tested the effect of the protease inhibitor aprotinin, on the catalytic activity of DENV-2 NS2b-NS3Pro enzyme. Aprotinin is a serine protease inhibitor which can bind NS2b-NS3 strongly [20], and inhibit it effectively at nanomolar concentrations [21]. Our data showed that aprotinin inhibited the recombinant protease activity effectively (IC_50_ = 20nM; Figure 1C).

### Compound MB21 inhibits DENV-2 NS2b-NS3Pro

**Figure 2:**
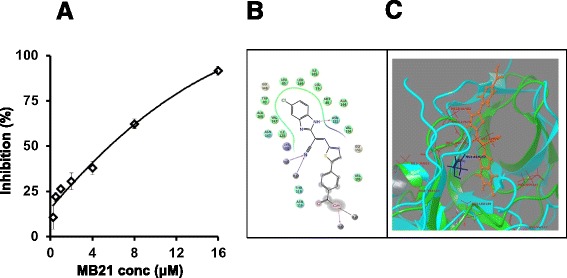
**Inhibition of DENV-2 NS2b-NS3Pro by MB21 and** ***in silico*** **analysis of the interaction between the two. (A)** Inhibition of protease activity of cloned DENV-2 NS2b-NS3Pro as a function of MB21 concentration. **(B)** Computer generated 2D ligand interaction picture depicting the interaction between MB21 and DENV-2 NS2b-NS3Pro. Hydrophobic residues are shown in green. **(C)** Interaction of MB21 at the allosteric pocket in the vicinity of the catalytic triad.

**Figure 3:**
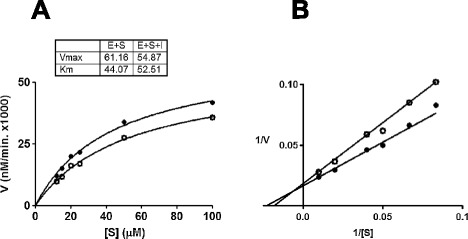
**Mode of inhibition of DENV-2 NS2b-NS3Pro by MB21. (A)** Enzymatic activity of DENV-2 NS2b-NS3Pro as a function of substrate concentration in the absence (filled circles) and presence of 6 μM MB21 (empty circles). The table shows kinetic parameters in presence (E + S + I) and absence (E + S) of MB21. E, S and I denote the enzyme, DENV-2 NS2b-NS3Pro, the substrate, Bz-nKRR-AMC, and the inhibitor, MB21. **(B)** Lineweaver-Burke plot of DENV-2 NS2b-NS3Pro activity in the absence (filled circles) and presence of 6 μM MB21 (empty circles). Data were analyzed using GraphPad Prism software.

With a functionally validated DENV NS2b-NS3pro assay in hand, we next proceeded to screen an ‘in-house’ library of ~1000 small molecular weight compounds to identify potential inhibitors. Recent work has shown that this library contains antimicrobial compounds [23,24]. An initial screen wherein these compounds were tested at a single concentration (25 μM), identified 25 compounds which manifested >80% inhibition of the recombinant NS2b-NS3Pro. One of these, a benzimidazole compound, MB21, was the most potent, manifesting an IC_50_ of 5.9 μM against the recombinant DENV-2 NS2b-NS3pro enzyme (Figure 2A). Three additional benzimidazole compounds, RB02, RA14 and RA16, also inhibited the cloned DENV-2 protease, albeit at comparatively lower efficiency (Additional file 1: Figure S3). We used *in silico* molecular docking to understand how MB21 may interact with DENV-2 NS2b-NS3Pro. This analysis showed that MB21 bound to the DENV- protease with its benzimidazole moiety embedded well within the hydrophobic cleft of an allosteric site [25], in the vicinity of the catalytic triad, as depicted in Figure 2 (panels B and C). Features of MB21 binding observed here correlate with earlier reports on allosteric binding [25,26]. To understand better the mechanism of action of MB21 on DENV-2 NS2b-NS3Pro, we determined the efficiency of protease action over a range of substrate concentrations in the absence and presence of varying MB21 concentrations (Figure 3A). These data were analyzed using Lineweaver-Burke double reciprocal plot (Figure 3B). We observed that both the kinetic parameters, *K*_*m*_ and *V*_*max*_, were changed by MB21. These data lead to the conclusion that MB21 may act by as a mixed inhibitor of DENV-2 NS2b-NS3Pro. This is consistent with the *in silico* docking data which show that MB21 binds to an allosteric site.

**Figure 4:**
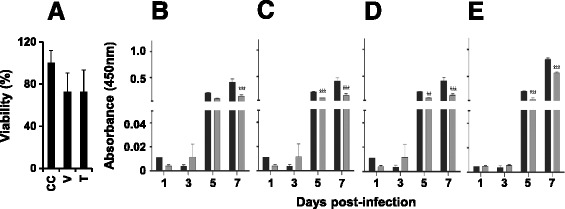
**Evaluation of antiviral activity using cell-based assay. (A)** Histogram showing the viability of Vero cells that received no drug (‘CC’, cell control), 1% DMSO vehicle alone (‘V’) or 10 μM MB21 in 1% DMSO vehicle (‘T’). Panels B-E depict the effect of MB21 on NS1 secretion by DENV-infected cells. Vero cells were infected with DENV-1 **(B)**, DENV-2 **(C)**, DENV-3 **(D)** or DENV-4 **(E)** either in the absence (black bars) or presence (grey bars) of MB21. Culture supernatants withdrawn at the indicated time points during the 1 week experiment, were tested for viral antigen levels using the Dengue NS1 ELISA kit (the NS1 ELISA absorbance scale on the Y-axis is the same for panels **B**-**E**). Data shown are mean values (n = 3). The vertical bars represent standard deviation, SD. Two-way ANOVA and Bonferroni post-test analysis was done using GraphPad Prism. *P* values were either significant (**) or very significant (***).

As a next step, we sought to assess if MB21 which inhibited cloned viral NS2b-NS3Pro efficiently had any inhibitory activity on the parent virus itself. To this end we used a cell-based assay in which we infected Vero cells with DENV in the presence of MB21 and monitored the effect of the drug on virus replication. However, before testing MB21 for its DENV inhibitory potential, we sought to ascertain if this compound manifested any cytotoxicity on Vero cells. Interestingly, we found that MB21 at concentrations up to 50 μM (in 0.5% DMSO vehicle) did not manifest any discernible cytotoxicity compared to controls (treated with 0.5% DMSO). This was essentially the case even at 100 μM MB21 (in 1% DMSO), as evidenced by comparable cell viability between 100 μM MB21-treated and 1% DMSO-treated cells, as shown in Figure 4A. It is to be noted, however, that 1% DMSO by itself, caused ~30% loss of cell viability, compared to cells that were not treated with DMSO. Based on these results, we conclude that MB21 up to 100 μM final concentration does not manifest discernible cytotoxicity on Vero cells.

It has been shown previously that DENV replication in cultured cells [27,28] and in animal models [29] can be monitored by determining the levels of the viral antigen NS1 using immunoassays. Recently we showed that the levels of NS1 antigen secreted into culture supernatants of infected cells closely mirror the viral genomic RNA levels, measured using quantitative RT-PCR, for all four DENV serotypes [27].

### MB21 is a pan-DENV inhibitor

Given the high degree of functional similarity of the NS2b-NS3Pro enzymes among the four DENV serotypes [20], we anticipated that MB21 may indeed be capable of inhibiting the replication of all four DENV serotypes. To test this, we infected Vero cells with each of the DENV serotypes separately, in the absence or presence of MB21 (at 30 μM final concentration), and monitored NS1 antigen synthesis as a marker of DENV replication. Culture supernatants were withdrawn at regular intervals over a 1 week period and analyzed for NS1 antigen levels using a commercially available ELISA kit. The data comparing the kinetics of viral NS1 antigen secretion, by each one of the DENV serotypes, into the culture supernatant in the absence and presence of MB21 are presented in Figure 4 (panels B to E). These results show that MB21 did indeed causes a statistically significant reduction of NS1 antigen levels secreted by all four DENV serotypes.

If NS1 secretion goes down, mirroring the down-regulation of viral replication, it follows that final viral titers must also be reduced in the presence of MB21. To ascertain this possibility, we measured DENV titers in a virus yield reduction assay. In this experiment, viral titers in DENV-infected culture supernatants (MB21 treated as well as untreated) harvested at day 3 post-infection were measured using a standard plaque assay (Additional file 1: Figure S4). It was seen that at all dilutions of the culture supernatant tested, the number of plaques in the presence of MB21 was significantly lower compared to that in the absence of the drug. This experiment was performed with all four DENV serotypes. The viral titers calculated from the plaque counts are summarized in Table 1. These data reveal that MB21 could inhibit DENV-1, −2, −3 and −4 titers by 50, 82, 75 and 73%, respectively. This was found to be statistically significant. The observed reduction in DENV titers correlates with the decrease in viral antigen synthesis (Figure 4, panels B-E) and corroborates NS1 as a marker for DENV replication.

**Table 1:** **DENV titers** ^***a***^ **(x10** ^**6**^ **pfu** ^***b***^ **/ml) in the absence and presence of MB21**

**DENV serotype** ^***c***^	**Without MB21**	**With MB21** ^***d***^	***P*** **value** ^***e***^
**1**	**1.06 ± 0.03**	**0.52 ± 0.02**	0.0028***
2	1.54 ± 0.12	0.27 ± 0.01	0.0045***
3	0.59 ± 0.01	0.14 ± 0.04	0.0065***
4	0.22 ± 0.02	0.05 ± 0.03	0.031**

^*a*^Titers were determined by plaque assay on Vero cells.

^*b*^Pfu = plaque forming units.

^*c*^The viral strains used were: DENV-1: West Pac 74; DENV-2: S-16803; DENV-3: CH54389; and DENV-4: TVP-360.

^*d*^MB21 used at 30 μM final concentration.

^*e*^ *P* values were calculated using Graphpad software; *P* values were either significant (**) or very significant (***).

Collectively, our data support the conclusion that MB21 is a pan-DENV inhibitor. The precise mechanism of the pan-DENV inhibitory activity of MB21 needs elucidation. Consistent with protease assay data, preliminary *in silico* docking (Figure 2) suggests that MB21 binds to the protease in the vicinity of its active site. The possibility that this binding may perturb the recently identified allosteric site (Ala125) on the protease [30] needs to be addressed. The likelihood that MB21 may compromise the ability of NS2b-NS3Pro to recruit fatty acid synthase during productive DENV infection [31] is another avenue to be explored.

## Conclusions

The need for dengue drugs is being increasingly felt as dengue vaccine continues to be elusive. Based on its critical role in the DENV life cycle, NS2b-NS3Pro has emerged as a potential antiviral target. We set up and validated an *in vitro* DENV protease assay and used it to initiate a random screening campaign to search an ‘in-house’ small molecule compound library, from which we have recently identified molecules with antimicrobial action [23,24], for putative pan-DENV inhibitor(s). One molecule from this library, a benzimidazole compound, MB21, was a potent inhibitor of the cloned DENV-2 protease, NS2b-NS3Pro (IC_50_ = 5.95 μM). It appeared to bind to an allosteric site in the vicinity of the active site. Examination of steady state enzyme kinetics followed by double reciprocal plot analysis indicated that MB21 affects both *K*_*m*_ and *V*_*max*_ and presumably functions as a mixed type inhibitor of NS2b-NS3Pro. Further it did not manifest significant cytotoxicity at concentrations as high as 100 μM. Interestingly, MB21 could suppress NS1 antigen secretion by all four DENVs, suggesting that it could function as a pan-DENV inhibitor. This was corroborated by plaque assay data which showed that viral titers were indeed reduced by MB21 in the case of each of the four DENV serotypes. The precise mechanism of action of MB21 on DENV replication needs to be elucidated. This molecule may provide a lead for further optimization.

## Methods

### Cells, viruses, reagents

The four DENV serotypes used in this study are the World Health Organization reference strains (DENV-1 West Pac 74, DENV-2 S-16803, DENV-3 CH54389 and DENV-4 TVP-360) and were kindly provided by Dr. De Silva, University of North Carolina, USA. The monkey kidney Vero cell line was from American Type Culture Collection, Virginia, USA. It was maintained in Dulbecco’s Modified Eagle medium (DMEM), supplemented with 10% (v/v) heat-inactivated (Δ) fetal calf serum (FCS), in a 10% CO_2_ humidified incubator, at 37°C.

The synthetic peptide substrate Bz-nKRR-MCA was custom-synthesized (Peptides International, Louisville, Kentucky, USA). NS1 ELISA kit was from J. Mitra & Co. Pvt Ltd, New Delhi, India. MTT (3-(4, 5-dimethylthiazolyl-2)-2, 5-diphenyltetrazolium bromide) assay kit was from Invitrogen (Life Technologies, USA).

The BITS in-house small molecule library consisted of diverse small molecules that included benzimidazoles, benzothiazoles, quinolones, thiazoles, thiazolidines, azetidines and spiropiperidones among others. Details of synthesis of the benzimidazoles identified as DENV-2 protease inhibitors are provided in Additional file 2.

### Protease and protease inhibition assays

Protease assays were carried out in 100 μl volume in microtiter wells of 96-well plates, essentially as described earlier [20]. A typical protease reaction (100 μl) contained 5nM purified DENV-2 rNS2b-NS3Pro enzyme (15 ng protein) in assay buffer (50 mM Tris–HCl, pH 8.5/1 mM CHAPS/20% glycerol). The reaction was initiated by the addition of peptide substrate Bz-nKRR-AMC (10 mM stock) to a final concentration of 10 μM. The reaction was incubated at 37°C for 20 minutes. Protease activity was measured in terms of the increase in fluorescence that accompanied cleavage of the peptide substrate (*λ*_ex_: 380 nm; *λ*_em_: 450 nm). Control reactions in which the protease was omitted were run in parallel to correct for background fluorescence of the substrate. To measure protease inhibition, the test compound was incorporated into the protease reaction prior to substrate addition. Enzyme control (EC) reactions set up in parallel contained an equivalent amount of the vehicle (DMSO) without any inhibitor. Half maximal inhibitory concentration (IC_50_) was defined as the inhibitor (test compound) concentration that decreased protease activity by 50%, with reference to the EC reaction (which was taken as 100%), under the experimental conditions. All assays were run in duplicates or triplicates and each experiment was performed at least twice independently.

### Vero cell-based DENV inhibition assay

The titers of the stock viruses used were as follows: DENV-1: 1.6×10^6^; DENV-2: 1.3×10^6^; DENV-3: 1.5×10^7^; & DENV-4: 1.5×10^9^ PFU/ml. Vero cells were seeded in 48-well plates (4×10^4^ cells/well in 0.5 ml DME + 10%ΔFCS) and incubated for 24 hours (37°C, 5% CO_2_). Monolayers were aspirated and treated with 0.5 ml DME + 0.5% with ΔFCS containing 30 μM final concentration of the test compound. After 1 hour incubation medium + test compound was removed and saved. The monolayer was infected with DENV (m.o.i = 0.1; 200 μl/well in DME + 0.5% with ΔFCS). After 2 hours, the virus inoculum was removed and replaced with medium containing test compound. The plate was returned to the incubator. Drug concentration was maintained by the addition of 5 μl stock compound (equivalent to 30 μM final concentration) solution into each well on days 3 and 5 post-infection. Aliquots (20 μl) of culture supernatant were withdrawn at indicated time points up to 7 days for estimation of NS1 antigen by ELISA and viral titers by plaque assay. Appropriate virus controls (VC) for each DENV serotype, wherein the drug treatment was omitted, were run in parallel. All infection experiments were done twice independently. Inhibition by a test compound was assessed with reference to VC which was taken to represent 100% infectivity.

### DENV NS1 determination

Culture supernatants collected at various time points, which were stored frozen at −20°C, were thawed and diluted appropriately (1:100 to 1:1000 in DME + 0.5%ΔFCS). Suitable aliquots (50 μl) of this were used to detect DENV NS1 antigen using a commercially available Dengue NS1 ELISA kit (J. Mitra & Co., India), as per the manufacturer’s protocol. This kit uses N- and C-terminal domain-specific anti-NS1 antibodies to detect the NS1 antigen produced by all four DENV serotypes.

### MTT assay

Vero cells were seeded in a 96-well microtiter plate (5,000cells in 200 μl DME + 5%∆ FBS) were exposed to different test compounds at a range of concentrations (2-100 μM) for four days at 37°C in a 10% CO_2_ incubator. Control wells received an equivalent amount of DMSO vehicle without the test compound. Cell viability was assessed based on the reduction of MTT using a commercial kit as per the manufacturer’s instructions.

### Statistical analysis

The statistical significance between MB21-treated and untreated samples was assessed using GraphPad Prism v6 for Windows. Differences were considered statistically significant when the probability levels (*P*) were <0.05.

### *In silico* studies

Molecular docking of MB21 onto the three-dimensional crystal structure of DENV-2 NS2b-NS3Pro (pdb code: 2FOM) obtained from the protein data bank (www.rcsb.org) was performed using GLIDE extra precision module (Glide v5.7, Schrodinger, LLC, New York, NY) as described [32].

## Additional files

Additional file 1:
**Supplementary results Figures S1-S4.**
Additional file 2:
**Synthesis of benzimidazoles.**
