# Supplementary material for: A small molecule inhibitor of dengue virus type 2 protease inhibits the replication of all four dengue virus serotypes in cell culture
Source: Virol J. 2015 Feb 8;12:16. doi: 10.1186/s12985-015-0248-x (PMC4327787; doi:10.1186/s12985-015-0248-x)
Supplement: Additional file 1: — Supplementary results Figures S1-S4. [file 12985_2015_248_MOESM1_ESM.docx]

**Figure S1: DENV-2 protease expression plasmid and predicted primary structure of rNS2b-NS3Pro enzyme.** (A) Map of the rNS2b-NS3Pro *E. coli* expression plasmid. The salient features of this plasmid include: the phage T7 promoter (pT7), *Lac* repressor gene (*Lac I*), Kanamycin marker (*Kan^R^*) and replication origin sequences (*Ori*). The synthetic *NS2b-NS3Pro* gene is inserted under the control of pT7. The chimeric gene consists of NS2b-derived sequences encoding the hydrophilic co-factor domain (red box) fused in frame to the protease-encoding domain of NS3 (blue box). These two are fused in frame through a non-cleavable peptide linker encoding sequence (grey box). The white box at the 3’ end denotes sequences encoding 6x His tag. (B) The amino acid sequence of the protein encoded by the r*NS2b-NS3* gene is shown. The different sequence components are indicated using the same colors as shown in panel A.

**Figure S2: *E.coli*-expression of DENV-2 rNS2b-NS3Pro and its affinity purification.** (A) SDS-PAGE analysis of rNS2b-NS3Pro expression following IPTG induction at 30^o^C. This panel displays the Coomassie-stained polypeptide profiles of total extracts prepared from un-induced (U) and induced (I) *E. coli* cells harboring the plasmid shown in Figure S1A. Lanes ‘P’ and ‘S’ display polypeptide profiles in the pellet and supernatant fractions, respectively, of induced cell extracts. (B) This panel shows polypeptide profiles obtained from P and S fractions of cells induced at 18^o^C. (C) Native Ni^2+^-NTA affinity purification of NS2b-NS3 from *E. coli* induced at 18^o^C. The panel shows the SDS-PAGE polypeptide profiles at different stages of the purification, which are indicated by the following abbreviations above the lanes: S (supernatant fraction from induced cell lysate), F (flow-through), W (20mM imidazole wash), E_1_ to E_5_ (fractions eluted using 100mM imidazole). Positions of the protein markers are shown to the left of the panel. (D) Immunoblot analysis of pooled fractions (r2b-3) from panel C using penta-His mAb. Pre-stained protein size markers were run in lanes marked ‘M’. Their sizes (in kDa) are indicated to the left of panel A. The arrow on the right of each panel indicates the position of the rNS2b-NS3 protein.


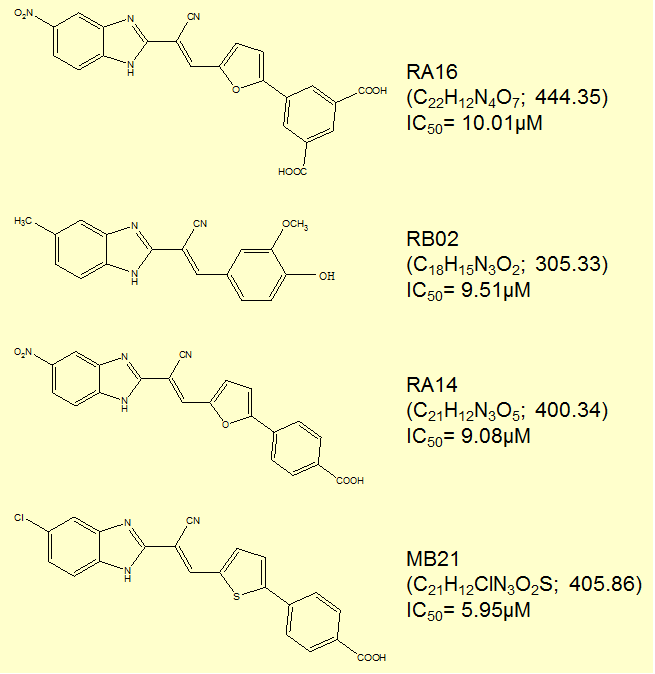


**Figure S3: Small molecule inhibitors of cloned DENV-2 NS2b-NS3Pro.** Shown to the right of each molecule is the name of the compound, its molecular formula and weight (in parenthesis) and the DENV-2 protease inhibitory potential (IC_50_ value). MTT assay showed that at 30µM concentration RB02 was cytotoxic to Vero cells, while the remaining three were not. When these latter molecules were tested for antiviral activity against DENV-2 in infected Vero cells, RA16 was without inhibitory effect, presumably because it failed to enter the cells. RA14 and MB21 inhibited DENV-2 by ~30% and ~70% respectively.

**Figure S4: The effect of MB21 on DENV replication.** Vero cells were infected separately with each of the four DENVs in the presence or absence of 30μM MB21. DENV titers in culture supernatants drawn on day-3 post-infection were determined by standard plaque assay. A. This panel shows a typical plaque assay experiment performed with different dilutions (10^-2^ to 10^-4^) of supernatants obtained from Vero cell cultures infected with DENV-4 in the absence (VC) or presence of MB21 (MB21). B. Histogram showing the percent inhibition of DENV-1 (D1), DENV-2 (D2), DENV-3 (D3) and DENV-4 (D4) titers in the presence of MB21 with reference to those in its absence (taken as representing 0% inhibition). Data shown are mean values (n=3). The vertical bars represent standard deviation, SD.
