# Supplementary material for: A small molecule inhibitor of dengue virus type 2 protease inhibits the replication of all four dengue virus serotypes in cell culture
Source: Virol J. 2015 Feb 8;12:16. doi: 10.1186/s12985-015-0248-x (PMC4327787; doi:10.1186/s12985-015-0248-x)
Supplement: Additional file 2: — Synthesis of benzimidazoles. [file 12985_2015_248_MOESM2_ESM.docx]

**Synthesis of benzimidazoles RB02, RA14, RA16 and MB21**

***Scheme***: The synthesis began by fusing the respective phenylenediamine with ethyl-2-cyanoacetate at 200^o^C. The so obtained 2-cyanomethylbenzimidazole derivative was then functionalized at the acetonitrile chain *via* Knoevenagel condensation with desired aldehyde in refluxing ethanol using piperidine as base (Figure S1). All the reactions progressed smoothly giving the titled acrylonitrile product in very good yield and purity. Both analytical and spectral data (^1^H NMR, ^13^C NMR and mass spectra) of all the synthesized compounds were in full agreement with the proposed structures.





***Figure S1.****Scheme of synthesis.*

***Experimental section****:* All commercially available chemicals and solvents were used without further purification. TLC experiments were performed on alumina-backed silica gel 40 F254 plates (Merck, Darmstadt, Germany). The homogeneity of the compounds was monitored by thin layer chromatography (TLC) on silica gel 40 F254 coated onto aluminum plates, visualized by UV light and KMnO_4_ treatment. Flash chromatography was performed on a Biotage Isolera apparatus with prepackaged disposable normal-phase silica columns. All ^1^H and ^13^CNMR spectra were recorded on a Bruker AM-300 (^1^HNMR: 300.12MHz; ^13^CNMR: 75.12MHz) NMR spectrometer (Bruker BioSpin Corp, Germany). Chemical shifts (*δ*) are reported in ppm with reference to the internal standard tetramethylsilane. The signals were designated as follows: br: broad; s: singlet; d: doublet; dd: doublet of doublets; t: triplet; m: multiplet. The molecular weights of the synthesized compounds were checked with an LCMS 6100B series instrument from Agilent Technology. Elemental analyses were carried out on an automatic Flash EA 1112 series CHN Analyzer (Thermo). The purity of the final compounds was examined by HPLC (Shimadzu, Japan; with a Phenomenex C8 (150×4.6mm, 5μm, 100Å) double-end-capped reversed-phase (RP) HPLC column) and was greater than 95%.

**General procedure for the synthesis of acrylonitrile derivatives**

To a warm solution of the corresponding 2-cyanomethylbenzimidazole (0.01 mol) in absolute ethanol (8 ml) was added the corresponding aldehyde (0.01 mol) and catalytic piperidine (0.003 mol). The reaction mixture was then stirred and heated to 80^o^C for 1-2 h, (as monitored by TLC and LCMS for completion), the precipitate formed was collected by suction and recrystallised from ethanol to give the desired product in good yield as mentioned in the Table S1 below.

**Table S1.** Properties of benzimidazole derivatives





| **Cmpd.** | **R** | **R_1_** | **Yield** | **Melting point** | **Molecular**  **formula** | **Molecular weight** |
| --- | --- | --- | --- | --- | --- | --- |
| **RB02** | CH_3_ |  | 79 | 286-288 | C_18_H_15_N_3_O_2_ | 305.331 |
| **RA14** | NO_2_ |  | 73 | 229-231 | C_21_H_12_N_3_O_5_ | 400.344 |
| **RA16** | NO_2_ |  | 67 | 287-289 | C_22_H_12_N_4_O_7_ | 444.354 |
| **MB21** | Cl |  | 76 | 277-279 | C_21_H_12_ClN_3_O_2_S | 405.857 |

**RB02:**Compound RB02, (E)-3-(4-hydroxy-3-methoxyphenyl)-2-(5-methyl-1H-benzo[d]imidazol-2-yl) acrylonitrile, was synthesized according to the above general procedure using 5-methyl-((2-benzimidazolyl) acetonitrile) (0.25g, 1.46 mmol), 4-hydroxy-3-methoxybenzaldehyde (0.22g, 1.46 mmol) and piperidine (0.037g, 0.44 mmol) to afford **RB02** (0.352g, 79%) as solid. M.p: 286-288 ^o^C. ^1^H NMR (CDCl_3_): *δ*_H_. 2.31(s, 3H), 3.86 (s, 3H), 7.06- 7.58 (m, 6H), 8.12 (s,1H). ^13^C NMR (CDCl_3_): *δ*c. 154.1, 149.2, 148.2, 141.3, 139.1, 136.2, 133.1, 129, 125.6, 122.5, 119, 116.5, 115.6, 115.3, 112, 107.6, 57.2, 21.6. ESI-MS *m*/*z* 306.1 (M+H)^+^. Anal Calcd for C_18_H_15_N_3_O_2_: C, 70.81; H, 4.95; N, 13.76; Found: C, 70.79; H, 4.94; N, 13.75.

**RA14:** Compound RA14, (E)-4-(5-(2-cyano-2-(5-nitro-1H-benzo[d]imidazol-2-yl) vinyl)furan-2-yl)benzoic acid, was synthesized according to the above general procedure using 5-nitro-((2-benzimidazolyl) acetonitrile) (0.25g, 1.24 mmol) and 4-(5-formylfuran-2-yl)benzoic acid (0.268g, 1.24 mmol), piperidine (0.032g, 0.37 mmol) to afford **RA14** (0.362g, 73%) as solid. M.p: 229-231^o^C. ^1^H NMR (CDCl_3_): *δ*_H._6.89 - 8.42 (m, 10H).^13^C NMR (CDCl_3_): *δ*c.171.2, 155.9, 150.3, 145.2, 144.8, 144.6, 141.8, 140, 135.6, 130.3, 127.3, 122.9, 119.1, 118.7, 116, 113.5, 113, 110.2, 106.3. ESI-MS *m*/*z* 401.2 (M+H)^+^. Anal Calcd for C_21_H_12_N_4_O_5_: C, 63; H, 3.02; N, 13.99; Found: C, 62.98; H, 3.01; N, 14.02.

**RA16:** Compound RA16, (E)-5-(5-(2-cyano-2-(5-nitro-1H-benzo[d]imidazol-2-yl) vinyl)furan-2-yl) isophthalic acid, was synthesized according to the above general procedure using 5-nitro-((2-benzimidazolyl) acetonitrile) (0.25g, 1.24 mmol), 5-(5-formylfuran-2-yl)isophthalic acid (0.32g, 1.24 mmol), piperidine (0.032g, 0.37 mmol) to afford **RA16** (0.369g, 67%) as solid. M.p: 287-289 ^o^C. ^1^H NMR (CDCl_3_): *δ*_H._ 6.87 – 8.53 (m, 7H), 8.95 (s, 2H).^13^C NMR (CDCl_3_): *δ*c.170.9, 156.1, 150.3, 145.1, 144.9, 144.6, 141.7, 140, 136.1, 130.8, 130.5, 130, 118.9, 118.6, 115.8, 113.5, 112.9, 110, 106.2. ESI-MS *m*/*z* 445.1 (M+H)^+^. Anal Calcd for C_22_H_12_N_4_O_7_: C, 59.47; H, 2.72; N, 12.61; Found: C, 59.48; H, 2.7; N, 12.59.

**MB21:** Compound MB21, (E)-4-(5-(2-(5-chloro-1H-benzo[d]imidazol-2-yl)-2-cyanovinyl) thiophen-2-yl) benzoicacid, was synthesized according to the above general procedure using 5-nitro-((2-benzimidazolyl) acetonitrile) (0.25g, 1.3 mmol), 4-(5-formylthiophen-2-yl)benzoic acid (0.3g, 1.3 mmol), piperidine (0.033g, 0.39 mmol) to afford **MB21** (0.403g, 76%) as solid. M.p: 277-279 ^o^C. ^1^H NMR (CDCl_3_): *δ*_H._ 7.21-8.09 (m, 10H).^13^C NMR (CDCl_3_): *δ*c.169.8, 143.2, 141.8, 141.6, 140.7, 140, 137.8, 136.9, 130.3, 130.1, 129.4, 128.7, 127.9, 127.6, 123.9, 118.6, 116.7, 116, 113.9. ESI-MS *m*/*z* 406.1 (M+H)^+^. Anal Calcd for C_21_H_12_ClN_3_O_2_S: C, 62.15; H, 2.98; N, 10.35; Found: C, 62.17; H, 3.01; N, 10.37.

## *******
